# Space Medicine
*This is a paper presented to the Cornwall Clinical Society on 6th January, 1956.


**Published:** 1956-07

**Authors:** S. G. Flavell Matts

**Affiliations:** Resident Medical Officer Royal Devon and Exeter Hospital Formerly Squadron Leader R.A.F.


### SPACE MEDICINE*

BY

S. G. FLAVELL MATTS, M.B., M.R.C.S.

*Resident Medical Officer Royal Devon and Exeter Hospital*
*Formerly Squadron Leader R.A.F.*

It may be felt that a discussion of this subject is a trifle premature at this time. However it is of interest to quote the words of Major-General H. G. Armstrong, U.S.A.F.,[1] referring to a similar discussion in 1952.

"The skill and ingenuity of the aeronautical engineer in designing aircraft with increased speed, endurance, and altitude performance, have currently projected the flying personnel into the immediate vicinity of an environmental frontier beyond which our knowledge is incomplete, and, in some respects, totally lacking. These gaps in our knowledge must be filled in at the earliest opportunity for at least the following reasons. A modern, high-performance military aircraft cannot be properly designed until the effect on the crew of the performance of that airplane and the environment through which it will fly is definitely known, because aircrew protective devices must be built-in design features of such an aircraft.

"This consideration points out a fact not generally recognized: that Aviation Medical Research, in this area at least, should be completed prior to the time the aeronautical engineer first sits down to his drawing-board, which is usually five to ten years before the projected airplane is first flown."

This illustrates the important and exciting part that medical science has to play in the conquest of space.

Over the past few years advances in science have brought the possibility of space travel within our grasp. However, there is little possibility of a space ship being able to make a successful trip without a human pilot. No computer which can cope with the multitude of problems liable to arise during take off and landing has yet been constructed; and it is quite impossible that one small enough to do this work could be built in the immediate future. Therefore, if we are to be successful, the space-ship will be manned by human beings. It is my purpose to point out to you some of the medical problems that will be encountered by the first voyager across the space frontier.

Firstly we have to face the fact that the human body was designed to operate efficiently on the surface of this earth. Obviously the putting of it into an unfamiliar environment will raise many problems. These may best be considered as they arise in a hypothetical "Journey into Space".

Before we "take off" I would like to clarify the position as regards the numerous so-called "barriers" that are frequently discussed in the popular press. By these I mean the so-called "sound", "heat" and "brain" barriers.

These are as realistic as the old idea that the human body could not travel faster than a hundred miles an hour. The only barriers that exist are due to gaps in our knowledge of conditions prevailing at various speeds. None of these need interfere in any way with our progression into space. Remember speed itself has no effect whatsoever on the human body; in fact all of us are travelling at a speed of a thousand miles per hour with the Earth's rotation! It is the change of speed, i.e. acceleration or deceleration, that brings the force of gravity to bear on the body and produces what are known as "*G* effects".

Now the "take off". In order to achieve 15,000 m.p.h., which is the minimum escape velocity, it will be necessary to accelerate very rapidly because of the rapid burning

* This is a paper presented to the Cornwall Clinical Society on 6th January, 1956.

of rocket fuel. This will inevitably produce a high-*G* loading on the pilot. This effect has been studied by using machines such as the man-carrying centrifuge, which simulates these high-*G* conditions. (There is one of these machines at the I.A.M. Farnborough and several in the U.S.A.). From these studies it is apparent that the effects of *G* on the body are dangerous mainly by their interference with the circulatory system. As the blood becomes heavier the load on the heart becomes greater and the circulation slows. The greater the *G* loading the slower the circulation becomes and pooling of blood in certain areas occurs. These are particularly the leg veins and abdominal veins. This pooling results in there being insufficient blood actually circulating. The amount of blood pooling is influenced by the position of the body relative to the line of resultant forces caused by change of velocity. Once the circulation has become greatly slowed, the brain, being the organ most sensitive to oxygen-lack, begins to stop working and dimness of vision, known as "grey out" and later loss of consciousness known as "black out" occurs. Another important factor is the extreme difficulty of movement under high-*G* conditions.

Our first step then will be to minimize the effects of *G*. This can be done in several ways, firstly by wearing a specially designed anti-*G* suit. This suit exerts its effect by pressing on the areas where blood pooling occurs and thus preventing this. It is automatically inflated when the *G* loading reaches a certain predetermined figure. This figure being found by testing beforehand in the centrifuge; and is the point at which the subject begins to notice visual or other disturbance. Secondly, the prone position is found to aid the circulation by reducing the height the blood has to be lifted, and increasing the *G* tolerance. Thirdly, the instruments will have to be power-operated, and very near the hands of the pilot. Fourthly the peak of physical fitness is essential.

Once the rocket has taken off it is necessary to provide a suitable atmosphere to breathe. The cabin will have to be pressurized to normal atmospheric conditions. The possibility of producing an artificial atmosphere by means of grasses in a so-called "botanical exchange unit" will not at present be possible, but may be essential later for longer trips. This would work because of the natural tendency of plants to absorb $CO_2$ and give off $O_2$; when irradiated with ultra-violet rays. Until more work has been done on this it seems likely that stored $O_2$ will have to be used. The pilot will not be wearing a space suit as this will be far too cumbersome for the wearer to carry out any intricate tasks. In fact he may be wearing nothing at all (in the absence of lady members of the crew) as there will be at first a considerable rise in the internal temperature of the cabin due to friction effects. (Additional active cooling plant may be necessary.)

What of the problem of a space suit? You may have seen pictures released by our American friends of a high-altitude or space suit designed for the U.S. Navy. Knowing their efficient security methods, you can be sure it is valueless! Otherwise such a detailed picture would not have been released. The problem is not so much to produce a suit which would be fully pressurized, as to produce one in which the individual can move about. A rigid suit could be constructed now, but would, of course, be useless. However, this is mainly an engineering problem and advances in flexible, tough materials are bringing the solution rapidly nearer.

Having reached the escape velocity we will begin to leave the region of the earth's gravitational field. The effect of gravity will rapidly decrease, as the force of gravity obeys the inverse square law of distance. Therefore we shall soon be under conditions of low or absent gravity and this will cause many physiological problems. The labyrinthine apparatus which normally regulates our sense of position and balance will start to present us with equivocal information, and it is possible that extreme nausea, vomiting, and vertigo may occur (as in labyrinthine disease). Also this will mean that to maintain orientation vision will be essential. In addition the blood pressure and speed of circulation will increase if the heart continues at its ordinary rate, for the speed of circulation is influenced by the stagnation effects in the veins produced by gravity.

Both these effects may be very serious and it seems likely that heavy medication will be necessary to slow the heart and to overcome the vertigo, &c.

Another serious problem will be eating and drinking. Drinking will be difficult because in the absence of gravity, fluid will not pour out of its container, and swallowing of solids is difficult because again there is no gravity to carry food to the back of the throat where the muscles of deglutition can take effect. Possible ways of overcoming this are by having both food and drink in capsules, or sucking through a tube.

Further, this lack of gravity will produce difficulty in eliminating faeces and urine, and extreme care will be necessary to avoid the spilling of them in the cabin! The possibility of having the space rocket spinning to provide a centrifugal "gravity" should not be considered likely at present, as it adds too greatly to the problem of controlling the rocket.

As soon as we leave the shielding effect of the atmosphere we shall be exposed to cosmic radiation. Much speculation has occurred about the dangers of cosmic rays. Now what is cosmic radiation? Mainly it consists of atomic nuclei freed from their surrounding electron rings.

These nuclei have only small powers of penetration of solids and it seems likely that the metallic space-ship walls will provide sufficient protection from the majority of them. However, there is a secondary danger because on collision with the metallic walls these nuclei will seize electrons from the atoms in the wall molecules, with the resultant release of small quantities of gamma radiation. Gamma radiation has great penetrating powers and is responsible for some of the dangerous effects of an atomic explosion; therefore if the quantities of gamma radiation are high it is likely that this effect will be extremely dangerous. Rocket experiments so far show that at a height of 200 miles this radiation is not serious and no serious genetic changes have been detected with prolonged similar doses in animal experiments.

What now of the psychological aspects? In every mind there dwells the elements of both claustrophobia and agorophobia. These will be magnified greatly by this voyage into the unknown. The cabin will be very small and there probably will be no direct vision ports, vision being achieved by radar and by periscope. When the pilot does look out through the periscope the vision of space as seen from outside the Earth's atmosphere is almost impossible to imagine! It is easy to say that the sky will be black and the stars bright; but the great impression will be the vastness of the universe, and the utter insignificance of man and his works in relation to this.

The question of so-called space sickness is at present undetermined. There seems no reason why it should occur. From our present knowledge of car, sea, and air sickness it seems that the factors are:

(1) a susceptible individual,

(2) unaccustomed motion,

(3) psychological factors varying from individual to individual.

In the absence of a susceptible individual there seems no way of producing motion sickness. Even bringing about complete disorientation by whirling a person round and round for some time cannot produce motion sickness in a resistant person. Pretesting to extreme limits would be essential before selecting the pilot.

In addition to these known syndromes, experimental work has indicated the possibility of a new syndrome brought about by interference with three of the basic facts to which the mind and body are accustomed:

(1) The instinct of survival, there being no escape from the rocket,

(2) The removal of the earth which the mind has always accepted as being near,

(3) Absence of gravity which the body has always experienced.

It may be necessary to provide hypnotic conditioning, and post-hypnotic commands may have to be instilled in the mind before it can accept these multiple changes of environment.

The final question is what is the danger of collision with a meteorite? Although this is not a medical question I would like to emphasize that calculations taking into account the relative size of the rocket, and the relative frequency of meteorites as counted by the radio-telescope, have shown this to be only a small danger.

So we are left with the thought that some day the vastness and splendour of the Universe may unfold before us like a beautiful garden, waiting to be explored by an enlightened race of men.

### REFERENCE

[1]Armstrong, H. G. (1952). *Physics and Medicine of the Upper Atmosphere* (Foreword). University of New Mexico Press.

